# Dexmedetomidine-Assisted Anesthetic Management of Empyema in a High-Risk Parturient With Cardiopulmonary Failure: A Case Report

**DOI:** 10.7759/cureus.83557

**Published:** 2025-05-06

**Authors:** Luke M Johnson, Steven Sartore, Dean Zhang, Jessica Potter

**Affiliations:** 1 Anesthesiology, Virginia Commonwealth University School of Medicine, Richmond, USA; 2 Anesthesiology, Virginia Commonwealth University, Richmond, USA

**Keywords:** ct guided drainage, dexmedetomidine, empyema, minimal sedation, one-lung ventilation (olv), video-assisted thoracoscopic surgery (vats)

## Abstract

Empyema, characterized by purulent fluid collection in the pleural space, presents treatment challenges. Video-assisted thoracoscopic surgery (VATS) and computed tomography (CT)-guided chest tube drainage are the main options, each with distinct anesthetic considerations. We describe the case of a 33-year-old pregnant woman (G8P8) at 29 weeks of gestation with a large left-sided empyema, cardiopulmonary failure, and a history of complex obstetric complications, including seven previous cesarean sections, placenta accreta, and placenta previa. After thorough multidisciplinary discussions and careful risk-benefit reviews, CT-guided chest tube drainage with monitored anesthesia care was chosen over VATS. The procedure was ultimately successful, emphasizing the need for individualized, multidisciplinary decision-making in critically ill pregnant patients. CT-guided drainage emerges as a viable alternative to VATS in select cases. Additionally, this case provides an example of using dexmedetomidine in a pregnant patient; this is significant as there is minimal research and reporting in this area of use.

## Introduction

Empyema is a collection of purulent fluid in the pleural space, most commonly caused by pneumonia [1]. While treatment options range in their level of invasiveness, the two most common procedural interventions are computed tomography (CT)-guided chest tube drainage and video-assisted thoracoscopic surgery (VATS) [1,2]. Deciding between these options can be challenging if patient factors do not offer a clear choice between them. CT-guided chest tube drainage offers better visualization in obese patients but sacrifices non-real-time views [3]. The key advantage of VATS for empyema is that it facilitates a swifter recovery for the patient [4]. Redden et al. directly compared the two above methods and found no statistically significant difference in mortality but did conclude that VATS may reduce length of hospital stay [5].

When considering anesthetic management, VATS is typically performed under general anesthesia, although it has been described in the literature that it can also be achieved with monitored anesthesia care (MAC) and a local anesthetic [6]. For CT-guided chest tube drainage, MAC is typically the most common anesthetic approach [3]. However, MAC may be challenging in patients who have difficulties lying flat, such as those with hypoxia, poor oxygenation status, or sleep apnea [7]. On the other hand, VATS requires careful preoperative evaluation, especially in patients with advanced cardiopulmonary disease, to ensure that one-lung ventilation (OLV), a common technique used during VATS, can be tolerated [6]. OLV involves deflating the operative lung near which the surgeon is working for better visualization, while maintaining ventilation of the other lung. This is typically achieved with a double-lumen endotracheal tube or a single-lumen endotracheal tube with a bronchial blocker [6].

Though both VATS and CT-guided chest tubes are safe during pregnancy [8,9], it is essential to consider the contrasting procedural settings in this patient population. While VATS takes place in the operating room (OR), CT-guided chest tube drainage typically occurs in a CT suite [5]. Thus, if a complication arose and urgent delivery was required, being in the operative theater for VATS would be highly preferred over the less-equipped CT suite, which lacks the resources of an OR (i.e., access to emergency airway equipment, medications, additional surgical staff, and monitoring) [5]. For the pregnant patient requiring image-guided chest tube placement, the anesthetic challenge includes striking the right balance of adequate sedation to maximize patient comfort while avoiding the over-sedation risks of fetal hypoxia/hypercarbia without the resources to safely intervene [10].

Dexmedetomidine is a highly selective alpha-2 adrenergic agonist that provides sedation, anxiolysis, and analgesia with minimal respiratory depression, making it a suitable option for procedural sedation in pregnant patients [11]. The drug's ability to maintain respiratory function is particularly advantageous in patients with respiratory conditions such as empyema [11].

The Society of Critical Care Medicine recommends dexmedetomidine for sedation in critically ill patients, highlighting its favorable safety profile [12]. The recommended infusion rate for dexmedetomidine in procedural sedation for adult patients is an initial loading dose of 1 mcg/kg over 10 minutes, followed by a maintenance infusion of 0.6 mcg/kg/hour, titrated to 0.2 to 1 mcg/kg/hour to achieve the desired level of sedation [13]. This dosing regimen should be carefully individualized to achieve adequate sedation while minimizing potential side effects such as bradycardia, hypotension, and sinus arrest [13].

Evidence from randomized controlled trials and case reports suggests that dexmedetomidine does not increase the risk of major birth defects or miscarriage when used during pregnancy, particularly in the second and third trimesters [13]. Most of these studies reported on dexmedetomidine usage during cesarean deliveries [13]. While the drug crosses the placenta, human studies have not demonstrated adverse effects at therapeutic doses [13]. Animal studies observed potential fetal toxicity, but only at significantly higher doses than those used in humans [13]. These effects occurred in rats given 1.8 times the maximum recommended human dose [13]. This substantial safety margin suggests the medication is unlikely to pose similar risks when used as directed during pregnancy. One animal study even suggested that dexmedetomidine has a potential neuroprotective role [14].

There are several important drug-drug interactions to consider when using dexmedetomidine. Co-administration of dexmedetomidine with anesthetics, sedatives, hypnotics, and opioids may lead to an enhancement of effects [13]. Research has confirmed these effects with sevoflurane, isoflurane, propofol, alfentanil, and midazolam [13]. Dexmedetomidine does not appear to impact the neuromuscular blockade associated with rocuronium in a clinically meaningful manner [13]. We present this case as an example of the beneficial application of dexmedetomidine in a high-risk parturient.

## Case presentation

**Figure 1:**
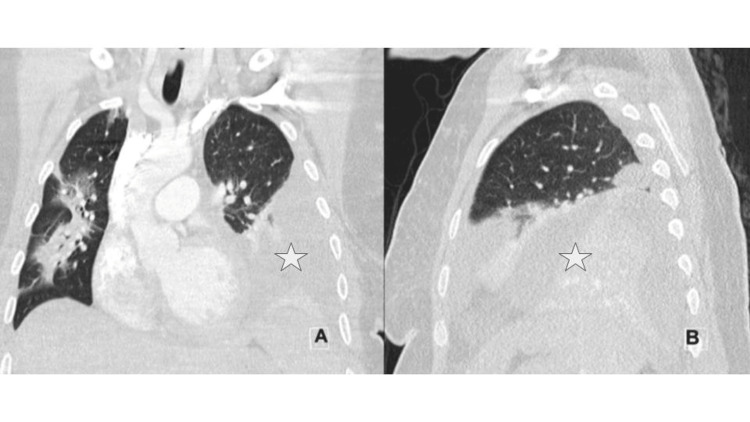
Computed tomography (CT) images showing a large left-sided empyema (white star) (A) Coronal view; (B) sagittal view

We present a 33-year-old (G8P8) female at 29 weeks gestation (BMI 35) who arrived at obstetric (OB) triage with coughing and emesis. Laboratory testing at the time was significant for human metapneumovirus (HMPV). The next day, she returned with worsening symptoms, including a productive cough, shortness of breath, and fever. She was diagnosed with superimposed *Streptococcus pneumoniae* infection, leading to septic shock and admission to the hospital’s medical intensive care unit (ICU). At the time of her ICU admission, she also presented with acute systolic heart failure, characterized by an ejection fraction (EF) of 40-45%. Ten days later, she was found to have a large left-sided empyema on chest CT (Figure 1). Her EF at this time had recovered to 55-60%.

In addition to her acute cardiopulmonary symptoms during this pregnancy, the patient had a complex OB history. She had a history of seven previous cesarean sections, one of which was notable for placenta previa. Her fourth pregnancy resulted in healthy twin deliveries, and the child of her seventh pregnancy was born with cerebral palsy. The patient’s current pregnancy was complicated by placenta accreta found on ultrasonography. With the potential for severe postpartum hemorrhage and the patient’s desire for sterility, she was planned to be delivered by cesarean hysterectomy.

The OB anesthesia team was involved in a multidisciplinary discussion regarding the best treatment path for the patient and her pregnancy. The two most viable options were a VATS or CT-guided chest tube with anterior and posterior drain placement using minimal sedation. Deciding between the options was challenging as the patient had legitimate considerations to account for with either procedure. Additionally, her physical exam - notable for baseline hypoxia, low cardiac reserve, shortness of breath, labored breathing, cough, and orthopnea - raised concerns about her ability to tolerate one option over the other. Initially, VATS seemed to be the preferred option as it would likely provide faster recovery for the upcoming cesarean hysterectomy and more complete drainage. VATS also would have eliminated the concerns about the patient having to lie flat without an established airway. However, there were significant concerns about using OLV and general anesthesia in a patient with cardiopulmonary compromise. Though the patient would have to be supine during CT-guided chest tube placement, risks could be mitigated with dexmedetomidine. Ultimately, both options were discussed with the patient, who opted for the more conservative, CT-guided chest tube option.

During the CT-guided chest tube placement, the proposed anesthetic plan centered around minimizing the aggressive hemodynamic changes associated with general anesthesia in this tenuous patient, while providing sufficient comfort to tolerate the invasive procedure. Medication selection was also tailored to minimize respiratory depression. A plan of MAC with sedation suited these goals while allowing the patient to preserve her own airway and respiratory status. Prior to the beginning of the case, the patient was placed on standard American Society of Anesthesiologists (ASA) monitors and positioned supine on the procedure table with a small ramp under her shoulders. Her supplemental oxygen via nasal cannula was proactively increased to 15 L/min for additional oxygenation. The sedation began with a bolus of 0.5 mg hydromorphone from the patient’s preexisting continuous ambulatory delivery device (CADD) and 2 mg of midazolam. As the case progressed, several boluses of dexmedetomidine and a single bolus of fentanyl were given for additional anxiolysis and analgesia. The total medications used were as follows: midazolam (2 mg), fentanyl (100 mcg), hydromorphone (0.5 mg), and dexmedetomidine (32 mcg). The patient tolerated the procedure well and maintained both oxygenation and spontaneous respirations throughout the case without the need for additional airway interventions or adjuncts. The case proceeded without complication; her supplemental oxygen was lowered to her previous baseline of 3 L/min via nasal cannula, and she was transported back to the ICU uneventfully.

On anesthesia post-evaluation, the cardiovascular and respiratory status was acceptable. The patient’s respiratory status improved from three liters of oxygen via nasal cannula with difficulty lying flat to room air within two days. Less than a month later, the patient had a cesarean hysterectomy at 34 weeks without any major complications or hemorrhage. Over a month after her cesarean hysterectomy, she was diagnosed with a pulmonary embolism in the emergency department and prescribed apixaban for treatment. She has not had any other medical concerns at the time of this writing.

## Discussion

This case highlights the unique and complex decision-making considerations involved in managing a pregnant patient with a significant OB history compounded by cardiopulmonary compromise and a large empyema. The choice between VATS and CT-guided chest tube drainage requires careful evaluation, especially when the patient’s profile does not clearly favor one option, as seen here. The patient’s history of multiple cesarean sections, placenta previa, and confirmed placenta accreta indicated a high-risk pregnancy. Additionally, her acute hypoxic respiratory failure and acute systolic heart failure, although improved at the time of the empyema diagnosis, further complicated her tenuous health status.

When considering VATS, the potential benefits included a faster recovery and more complete drainage, which could have been advantageous for the patient’s unborn child. However, VATS carries significant risks, as it typically requires general anesthesia and OLV. Given the patient's unstable cardiopulmonary status, the risk of compromise during OLV was a major concern. Additionally, with a planned cesarean hysterectomy, it was crucial to assess the implications of an emergent delivery during VATS. In the event of acute deterioration requiring an urgent cesarean hysterectomy, the anesthesia team would need the full resources of an OR for resuscitation while the OB team managed the surgical intervention. However, even in the OR, responding to complications would be challenging due to the positioning of surgical instruments during VATS.

On the other hand, CT-guided chest tube drainage offered the chance to minimize risks associated with general anesthesia, OLV, and surgical interventions. However, there were notable concerns with this approach as well. The patient's respiratory state made lying flat challenging, and the CT suite was a suboptimal setting in the event that an emergency procedure, such as an immediate cesarean-hysterectomy delivery, was needed should the fetus decompensate. The radiation exposure from the CT scan was another concern. While radiation exposure is typically minimized during pregnancy, the benefits generally outweigh the risks when the procedure is critical for maternal and fetal health, as was the case here [15]. Further, fetal doses from this procedure are typically well below the threshold associated with adverse fetal outcomes [16].

Both options were thoroughly evaluated in a multidisciplinary discussion involving the OB, thoracic surgery, and OB anesthesia teams. Ultimately, the decision was made to proceed with CT-guided chest tube drainage to minimize her exposure to the risks associated with general anesthesia and OLV, despite the associated challenges of intervening if the fetus becomes unstable.

Another anesthetic option that may be considered in empyema treatment is thoracic epidural anesthesia (TEA) [17]. Benefits of TEA in treating empyemas include maintaining postoperative analgesia [17]. Further, if urgent delivery had been necessary, having an epidural catheter already placed would be beneficial. However, TEA is associated with increased bleeding [17] and hypotension [18], which could be problematic in a patient with recent cardiac compromise and sepsis, as seen in this case.

This case highlights the role of dexmedetomidine as a valuable tool in the anesthetic management of high-risk parturients with cardiopulmonary compromise. Dexmedetomidine provided anxiolysis and sedation while minimizing the risk of hypoventilation, which would have been problematic for this patient due to her pulmonary status. However, the use of dexmedetomidine in pregnancy is not widely reported or well-studied, and more research and case studies are needed to further assess its safety and efficacy in this population. In this case, dexmedetomidine was likely beneficial, demonstrating that with careful monitoring, it may be a useful option in situations where respiratory preservation is critical.

## Conclusions

This unique case without a clear treatment-decision route demonstrates the complexity of managing a pregnant patient with significant cardiopulmonary compromise and a large empyema. CT-guided chest tubes avoid the potential complications associated with general anesthesia and OLV in VATS, but are complicated by orthopnea. In this instance, dexmedetomidine proved to be a viable sedative agent in an OB patient with pulmonary compromise.
